# Comparison of Postoperative Gastrointestinal Motility of Sugammadex and Neostigmine in Patients Undergoing Robotic Thyroidectomy: A Retrospective Study

**DOI:** 10.3390/jcm11102930

**Published:** 2022-05-22

**Authors:** Min Jeong Lee, Duk-Hee Chun, Hee Jung Kong, Hye Jung Shin, Sunmo Yang, Na Young Kim

**Affiliations:** 1 Department of Anesthesiology and Pain Medicine, Anesthesia and Pain Research Institute, Yonsei University College of Medicine, 50-1 Yonsei-ro, Seodaemun-gu, Seoul 03722, Korea; mmode@yuhs.ac (M.J.L.); sarajo8768@yuhs.ac (H.J.K.); sunmong@yuhs.ac (S.Y.); 2 Department of Anesthesiology and Pain Medicine, CHA Bundang Medical Center, CHA University, Seongnam 13496, Korea; leah1013@cha.ac.kr; 3 Biostatistics Collaboration Unit, Yonsei University College of Medicine, 50-1 Yonsei-ro, Seodaemun-gu, Seoul 03722, Korea; hjshin105@yuhs.ac

**Keywords:** postoperative bowel movement, robotic thyroidectomy, sugammadex, neostigmine/glycopyrrolate, retrospective study

## Abstract

Postoperative bowel dysfunction poses difficulty to patients during their recovery from surgery, and reversal agents may affect bowel function. This study aimed to investigate and compare the effects of sugammadex and a neostigmine/glycopyrrolate combination on postoperative bowel movement in patients undergoing robotic thyroidectomy. The electronic medical records of 122 patients, who underwent robotic thyroidectomy between March 2018 and December 2020, were retrospectively reviewed. Demographic, clinical, and laboratory findings and the first gas-passing time after surgery were assessed. The number of patients with a first gas emission time over 24 h was significantly higher in the neostigmine group than in the sugammadex group (*p* = 0.008). Multivariate logistic regression analysis indicated that sugammadex was a prognostic factor for the first gas-passing time within 24 h (odds ratio = 4.60, 95% confidence interval 1.47–14.36, *p* = 0.005). Although postoperative bowel motility, based on the first gas emission time, was comparable, the number of patients with a first gas emission time within 24 h was significantly higher in the sugammadex group than in the neostigmine group. This shows that the use of sugammadex did not affect the delayed recovery of postoperative bowel motility after robotic thyroidectomy.

## 1. Introduction

Acetylcholinesterase inhibitors (AChEIs), such as neostigmine or pyridostigmine, are used as reversal agents for neuromuscular (NM) blockade during general anesthesia. These compounds are known to enhance intestinal motility [1], but require anticholinergic drug co-administration to reduce muscarinic-mediated bradycardia and secretion production [2,3].

Recently, sugammadex has been applied for reversing aminosteroid NM blockade through encapsulation. Having no muscarinic activity, it does not require anticholinergic drug co-administration. Several studies have reported that using sugammadex for NM blockade reversal resulted in fewer respiratory complications and less residual NM blockade compared to the conventionally used AChEIs accompanied with anticholinergic agents, and that it contributed to enhanced recovery after surgery [4,5]. However, only a few studies have investigated the influence of sugammadex on postoperative bowel movements, despite the fact that an easily overlooked factor, potentially affecting postoperative bowel function, is the use of reversal agents of NM blockade during surgery. Moreover, its effects have not been clearly compared with those of AChEIs/anticholinergics.

A study by Deljou et al. [6] found that patients undergoing intraperitoneal surgery, who received sugammadex, had earlier first postoperative bowel movement compared to those who received neostigmine/glycopyrrolate. In addition, a faster return of bowel movements after colorectal surgery and open pancreaticoduodenectomy was observed with sugammadex compared to neostigmine/glycopyrrolate [7,8]. The factors affecting postoperative bowel dysfunction are secondary bowel dysfunction from operative trauma [9], surgical stress response [10], and opioid analgesics [11]. Therefore, it is important to eliminate bowel manipulating surgery to clearly evaluate the effect of sugammadex on postoperative bowel movement.

Thus, this study investigated the effects of sugammadex, compared with neostigmine/glycopyrrolate, on postoperative bowel motility in patients undergoing robotic thyroidectomy.

## 2. Materials and Methods

### 2.1. Study Population

**Figure 1:**
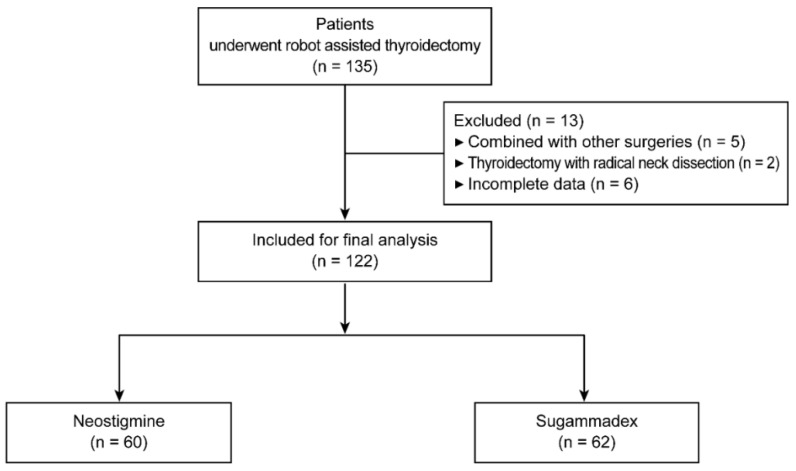
Flow diagram of patient selection.

This retrospective study was performed at a single institution in accordance with the ethical standards of the current version of the Declaration of Helsinki, after receiving approval from the Institutional Review Board and Hospital Research Ethics Committee (Yonsei University Health System, Seoul, Korea; protocol No. 4-2021-0134). All records of the patients were anonymized before the analysis, and obtaining informed consent from the patients was waived due to the retrospective nature of the study. The electronic medical records of 135 patients who were over the age of 20 years, with recorded gas emission times, and who underwent robotic thyroidectomy between March 2018 and December 2020, were assessed. Of the 135 eligible patients, five patients who underwent combined surgeries, two who underwent thyroidectomy combined with radical neck dissection following intraoperative frozen biopsy, and six with incomplete data were excluded. Finally, 122 patients were classified into two groups according to the reversing agents: patients who received sugammadex (*n* = 62) or neostigmine (*n* = 60) (Figure 1).

### 2.2. Clinical Practice

In the operating room, routine noninvasive blood pressure monitoring, electrocardiography, pulse oximetry, bispectral spectrometry, and a peripheral nerve stimulator to monitor NM blockade were performed. Anesthesia was induced with propofol (1.0–1.5 mg/kg) and remifentanil (0.05–0.1 μg/kg), and 0.6 mg/kg of rocuronium was injected thereafter, followed by endotracheal intubation. Anesthesia was maintained with an age-adjusted minimal alveolar concentration of end-tidal sevoflurane or desflurane of 0.8–1.2 combined with remifentanil infusion at a rate of 0.05–0.2 μg/kg/min to achieve a bispectral index score of 40–60. Based on the surgical condition, an additional 5–10 mg of rocuronium was administered to maintain a train-of-four count of 1–2 during surgery, according to the attending anesthesiologist. Robotic thyroidectomy was performed using the da Vinci Xi surgical robotic system (Intuitive Surgical, Sunnyvale, CA, USA) with the transaxillary approach [12]. Before the end of the surgery, propacetamol (1 g) or oxycodone (0.5 mg/kg) was administered to relieve postoperative pain. Concurrently, ramosetron (0.3 mg) was administered to reduce postoperative nausea and vomiting. Decisions regarding the use of agents to reverse NM blockade (neostigmine (1 mg) combined with glycopyrrolate (0.2 mg) or sugammadex (2 mg/kg)) were made by the attending anesthesiologist. When the patient recovered complete spontaneous respiration and consciousness, extubation was performed, and the patient was transferred to the post-anesthetic care unit (PACU).

### 2.3. Data Collection and Outcomes

The demographic data, including age, sex, body mass index (BMI), American Society of Anesthesiologists (ASA) physical status classification, and underlying diseases (hypertension, diabetes mellitus, and liver diseases) were assessed. The following operative data were evaluated: anesthesia time, anesthetic agents, dosage of the NM blocking agent, analgesic agent, total fluid input, type of thyroidectomy, and duration of PACU stay. Laboratory values such as thyroid-stimulating hormone (TSH), thyroid hormone, and serum calcium (Ca), as well as postoperative bowel motility and length of postoperative hospital stay, were collected.

The primary endpoint was the comparison of postoperative bowel motility based on the first gas emission time between the groups. First gas emission time was defined as the time from the end of anesthesia to the time of the first gas emission. Additionally, we investigated prognostic factors for the gas emission time, within 24 h after robotic thyroidectomy, as the secondary endpoint.

### 2.4. Statistical Analysis

Continuous variables are presented as mean ± standard deviation following an evaluation using the independent t-test, while categorical values are presented as the number and percentage of patients following the analysis with the chi-square test or Fisher’s exact test. Logistic regression analysis was employed to identify the prognostic factors for the gas-passing time, within 24 h after robotic thyroidectomy. All statistical analyses were performed using SAS version 9.4 (SAS Institute, Cary, NC, USA) software, and *p*-values <0.05 were considered statistically significant.

## 3. Results

The demographic characteristics of the patients are presented in Table 1. No significant differences were observed between the two groups receiving neostigmine/glycopyrrolate or sugammadex, in terms of the patients’ age, sex, BMI, co-morbidities, or preoperative laboratory values, including the levels of thyroid hormone, TSH, and serum Ca.

In addition, no operative variables differed between the two groups, other than the number of patients who received a rescue opioid during surgery, which was significantly higher in the sugammadex group compared with the neostigmine group (*p* = 0.031) (Table 2).

**Figure 2:**
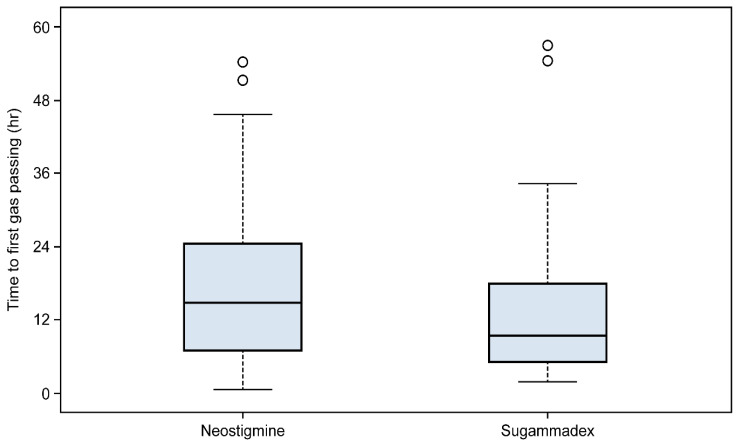
Distribution of the Wilcoxon scores for gas-passing time (h) after the operation in the neostigmine group (*n* = 62) and sugammadex group (*n* = 46).

The median gas-passing time was 14.8 (6.9–24.6 h) in the neostigmine group and 9.4 (5.2–17.8 h) in the sugammadex group, and no significant difference between the two groups was observed (*p* = 0.085, Figure 2). However, the number of patients with a first gas emission time over 24 h was significantly higher in the neostigmine group than that in the sugammadex group (*p* = 0.008, Table 3).

Table 4 shows the univariate and multivariate logistic regression analyses of prognostic factors for the first gas emission time within 24 h after robotic thyroidectomy. Following univariate analysis, sugammadex was significantly associated with the first gas-passing time within 24 h (odds ratio [OR] = 3.69, 95% confidence interval [CI] 1.34–10.15, *p* = 0.012) compared with neostigmine, and no other variables had a significant impact. Further, multivariate logistic regression analysis indicated that sugammadex was a prognostic factor for the first gas-passing time within 24 h (OR = 4.60, 95% CI 1.47–14.36, *p* = 0.005).

## 4. Discussion

This retrospective study demonstrated that postoperative bowel motility, based on the first gas emission time, was comparable between the sugammadex and neostigmine/glycopyrrolate combination groups. However, the number of patients with a first gas emission time over 24 h was significantly higher in the neostigmine group than in the sugammadex group, and multivariate logistic regression analysis revealed that sugammadex was significantly associated with the first emission time within 24 h after robotic thyroidectomy.

The muscarinic properties of AChEIs can promote bowel activity [13,14], particularly neostigmine, which is known to be effective in the treatment of ileus and pseudo-obstruction [15,16,17]. Since postoperative ileus is one of the most common complications after general anesthesia, especially for patients undergoing intraperitoneal surgery [18], neostigmine, administered to reverse NM blockade, can enhance the bowel movement that accelerates postoperative gas passing. However, co-administered anticholinergic agents that reduce the undesirable effects of AChEI, such as bradycardia, heart block, and arrhythmia, could prevent the AChEI’s effect on bowel movements [19]. As a reversal non-depolarizing NM blocking agent, sugammadex causes a minimal effect on gastrointestinal motility and is, therefore, expected to show a slower recovery time for postoperative bowel movements compared with AChEI administration. However, previous comparative reports on postoperative bowel function following the administration of sugammadex or AChEI with anticholinergic reported either no significant difference or earlier first flatus in the sugammadex-treated group [6,7,8,20,21,22].

In the current study, the median gas-passing time was 14.8 (6.9–24.6 h) in the neostigmine group and 9.4 (5.2–17.8 h) in the sugammadex group, which demonstrated no statistical difference between the sugammadex and neostigmine/glycopyrrolate combination groups (*p* = 0.085). However, the number of patients who did not release gas within 24 h after surgery was approximately three times higher in the neostigmine group than in the sugammadex group (*p* = 0.008). Furthermore, multivariate logistic analysis of prognostic factors for the gas emission time within 24 h after robotic thyroidectomy revealed that the OR of the sugammadex group was 4.6, which showed a significantly greater effect than that of the neostigmine group (*p* = 0.005).

The first study to evaluate the differences in first flatus times between sugammadex and neostigmine was completed by Sen et al. [20], where sugammadex was not associated with an improvement in postoperative bowel motility in patients undergoing open thyroidectomy. However, recent retrospective studies reported that the use of sugammadex was associated with earlier first postoperative bowel movement than the combination of neostigmine and glycopyrrolate in patients undergoing intraperitoneal surgery, pancreaticoduodenectomy, and colorectal surgery [6,7,8]. Our findings are consistent with those of recent studies that demonstrated that sugammadex was associated with a decrease in the occurrence of a prolonged time to postoperative bowel motility recovery [6,7,8]. However, while our study did not show any significant differences in gas-passing time between the sugammadex and neostigmine/glycopyrrolate groups, a significant difference between the groups was observed in previous studies. This discrepancy may be due to the lack of bowel manipulation during surgery, different doses, uses of AChEIs and anticholinergic agents, and the combination of other anticholinergic agents, such as atropine.

Factors commonly associated with postoperative gastrointestinal tract dysfunction include opioid analgesics, surgical stress responses, and bowel dysfunction secondary to operative trauma [9,10,11]. In the current study, univariate logistic regression indicated that the first gas-passing time within 24 h was not associated with the intraoperative use of opioids. Furthermore, even though the number of patients who received opioids during surgery was significantly higher in the sugammadex group than in the neostigmine/glycopyrrolate group, the sugammadex group showed an earlier gas-passing time. This is probably due to the amount of administered opioid, which was not enough to affect postoperative bowel movement.

In addition, post-orthopedic, bowel, or pelvic surgeries are prone to causing intestinal pseudo-obstruction [16,23]. The difference between intraperitoneal and non-intraperitoneal surgery lies in the manipulation of the bowel during surgery. In intraperitoneal surgery, sugammadex seems to accelerate bowel movement [6,7,8,21], but in non-intraperitoneal surgery, such as thyroidectomy, sugammadex and neostigmine/glycopyrrolate did not show significant differences in bowel movement [20]. However, according to a recent study by Deljou et al. [22], sugammadex resulted in earlier bowel function recovery in patients who underwent craniotomy; this is the first report to show that sugammadex was more effective for postoperative bowel motility than neostigmine/glycopyrrolate, even in non-intraperitoneal surgery. Along with this previous study, the current study can be used as the basis for future prospective studies focusing on the effectiveness of sugammadex on postoperative bowel motility following non-peritoneal surgery.

Postoperative ileus is an abnormal pattern of gastrointestinal motility, which occurs most often after abdominal surgery. Delayed gas passing, nausea, and vomiting have been reported as some of the features of postoperative ileus [18]. Although neostigmine has been implicated in an increased incidence of PONV, as a result of acting on the cholinergic receptor in the emetic center of the brain [24,25,26], different results were found depending on the dose of neostigmine administered [27,28]. In previous studies, PONV was evaluated as a secondary endpoint accompanied with postoperative bowel movement, but no differences were shown between the groups [7,8]. Due to the high incidence rate of PONV after thyroidectomy [29], all patients received antiemetics in the admission room. Thus, only the number of patients who required additional rescue antiemetics in the PACU were evaluated, where no difference between the groups was observed. This retrospective nature is the main drawback of the current study. However, based on the results of this retrospective study, further prospective controlled studies evaluating PONV concomitant with postoperative bowel motility will have much more clinical significance.

Another limitation of the present study is its small sample size, obtained from patients who underwent robotic thyroidectomy, where the incidence of postoperative ileus is not frequent. However, it could be strongly argued that because the influence of surgical manipulation on the intestines can be readily excluded, choosing patients who only underwent robotic thyroidectomy strengthens the validity of our results and allows for a more accurate comparison of the two agents’ effects on bowel movement. Additionally, in the sugammadex group, the dosage of sugammadex was adjusted according to weight, while in the neostigmine group, 1 mg of neostigmine and 0.2 mg of glycopyrrolate were used for all patients. Our use of a constant dosage of neostigmine and glycopyrrolate should be considered as one of the limitations of the current study. Moreover, although there are multiple variables to evaluate gastrointestinal motility, such as bowel sound, nausea, and vomiting, only the first gas-passing time was used to represent the gastrointestinal transit time, as this was the only available information from the electronic medical database. In addition, the paracetamol absorption or the colonic scintigram method would yield a more reliable measure of the transit time. To overcome these limitations and clarify the findings of this study, further large-scale prospective controlled trials are needed. Nonetheless, our findings are clinically meaningful to the existing literature.

## 5. Conclusions

Postoperative bowel motility based on the first gas emission time was comparable between the two groups; however, the number of patients with a first gas emission time within 24 h was significantly higher in the sugammadex group than that in the neostigmine/glycopyrrolate group. Furthermore, the multivariate logistic regression analysis revealed that sugammadex was significantly associated with the first emission time within 24 h after robotic thyroidectomy, suggesting that the use of sugammadex did not affect the delayed recovery of postoperative bowel motility after robotic thyroidectomy. However, further randomized controlled trials in different surgical contexts are required.

## Figures and Tables

**Table 1:** Demographic characteristics of patients.

Variables	Neostigmine (*n* = 60)	Sugammadex (*n* = 62)	*p*-Value
Age, years	39 ± 10	37 ± 10	0.135
Female sex	55 (92)	60 (97)	0.269
Body mass index, kg m^−2^	22.5 ± 3.2	23.1 ± 3.7	0.629
ASA physical status			0.592
I	46 (77)	50 (81)	
II	14 (23)	12 (19)	
Co-morbidities			
Hypertension	5 (8)	1 (2)	0.111
Diabetes mellitus	1 (2)	0 (0)	0.492
Liver disease	2 (3)	1 (2)	0.616
Preoperative laboratory value			
T3, pg/mL	0.90 ± 0.13	0.96 ± 0.20	0.065
T4, ng/dL	1.09 ± 0.22	1.09 ± 0.17	0.521
TSH, mIU/L	1.67 ± 1.11	1.49 ± 0.92	0.538
Serum calcium, mg/dL	9.51 ± 0.32	9.54 ± 0.31	0.580

Values are presented as mean ± standard deviation or number of patients (%). ASA, American Society of Anesthesiologists; T3, triiodothyronine hormone; T4, thyroxine hormone; TSH, thyroid-stimulating hormone.

**Table 2:** Perioperative variables.

Variables	Neostigmine (*n* = 60)	Sugammadex (*n* = 62)	*p*-Value
Duration of anesthesia, min	149 ± 39	140 ± 31	0.120
Sevoflurane/desflurane	3/57 (5/95)	4/58 (6/94)	>0.999
Intraoperative administered remifentanil, µg	494 ± 173	471 ± 171	0.472
Intraoperative administered rocuronium, mg	55 ± 8	53 ± 13	0.287
Intraoperative use of opioid, n	15 (25)	27 (44)	0.031 *
Intraoperative total fluid intake, mL	608 ± 154	608 ± 173	0.985
Subtotal/total thyroidectomy	50/10 (83/17)	54/8 (87/13)	0.558
Duration of PACU stay, min	37 ± 11	35 ± 9	0.193
Additional antiemetics in PACU, n	2 (3)	7 (11)	0.164
Serum calcium at POD 1, mg/dL	8.69 ± 0.36	8.72 ± 0.41	0.529
Hypocalcemia at POD 1, n	10 (22)	9 (21)	0.883
Length of postoperative hospital stays, days	3.1 ± 0.3	3.0 ± 0.3	0.363

Values are presented as mean ± standard deviation or number of patients (%). POD, postoperative day; PACU, post-anesthesia care unit. * *p* < 0.05.

**Table 3:** Postoperative bowel motility.

Variables	Neostigmine (*n* = 60)	Sugammadex (*n* = 62)	*p*-Value
Patients who passed gas > 12 h	35 (58)	26 (42)	0.070
Patients who passed gas ≤ 12 h	25 (42)	36 (58)	
Patients who passed gas > 24 h	17 (28)	6 (10)	0.008 *
Patients who passed gas ≤ 24 h	43 (72)	56 (90)	
Postoperative constipation	16 (27)	9 (15)	0.097

Values are presented as number of patients (%). * *p* < 0.05

**Table 4:** Univariate and multivariate logistic regression analyses of the prognostic factors for the gas emission time within 24 h after robotic thyroidectomy (*n* = 122).

Variables	Univariate		Multivariate	
	OR (95% CI)	*p*-Value	OR (95% CI)	*p*-Value
Group				
Neostigmine	1		1	
Sugammadex	3.69 (1.34–10.15)	0.012 *	4.60 (1.59–13.30)	0.005 *
Female sex	0.71 (0.08–6.16)	0.752		
Age, year	0.99 (0.94–1.03)	0.520		
Body mass index, kg m^−2^	1.12 (0.96–1.30)	0.141		
ASA physical status				
I	1			
II	0.55 (0.23–1.34)	0.188		
Anesthetic agent				
Sevoflurane	1			
Desflurane	0.74 (0.52–3.44)	0.698		
Remifentanil, 10 µg increase	1.03 (0.99–1.06)	0.115	1.03 (1.00–1.07)	0.094
Intraoperative use of opioid	0.78 (0.31–1.98)	0.599	0.48 (0.17–1.36)	0.169
Rocuronium, 1 mg increase	1.02 (0.98–1.07)	0.379		
Type of thyroidectomy				
Subtotal	1			
Total	2.02 (0.43–9.50)	0.371		
Total fluid intake, 100 mL increase	1.08 (0.81–1.44)	0.591		
Duration of anesthesia, 5 min increase	1.00 (0.93–1.06)	0.904		
Duration of PACU stay, 5 min increase	1.02 (0.98–1.06)	0.376		

OR, odds ratio; CI, confidence interval; ASA, American Society of Anesthesiologists; PACU, post-anesthesia care unit. * *p* < 0.05.

## Data Availability

The datasets used and/or analyzed during the current study are available from the corresponding author on reasonable request.
